# The Hahnemann Association of New York

**Published:** 1896-01

**Authors:** 


### THE HAHNEMANN ASSOCIATION OF NEW YORK.

The second annual meeting of the Hahnemann Association was very successful, being held at that well-known resort for good dinners, Delmonico's, November 21st, 1895.

An assemblage of ladies and gentlemen, filling Delmonico's large banquet hall, was present from New York and vicinity, with a number of members and visitors from Boston, Philadelphia and other places.

The social part of the meeting before the banquet was exceptionally successful, and every one enjoyed a pleasant hour of social converse. After a most excellent dinner, the President, Dr. J. Lester Keep, in calling the meeting to order, gave in a short address the history and aims of the Association, to not only honor the memory of Samuel Hahnemann, but to advance the interests of Homœopathy, by interesting and associating laymen and physicians in a body which can make its influence felt. Dr. F. J. Nott, in a very happy manner, filled the post of toast-master and introduced the following speakers:

Senator J. H. Gallinger, of New Hampshire; Hon. D. H. Chamberlain, ex-Governor of South Carolina; Hon. W. H. McElroy, and Rev. H. A. Brown, D. D.

Dr. Pemberton Dudley made a few remarks, and wished the Association success on behalf of the American Institute of Homœopathy, particularly in this coming centennial year.

A short address brought the meeting to a close.

The following officers were elected:

President, Naesteri Desebere, M. D.; First Vice-President, F. J. Nott, M.D.; Second Vice-President, C. W. Butler, M.D.; Third Vice-President, C. S. Macy, M.D.; Recording Secretary, S. H. Vehslage, M.D.; Corresponding Secretary, H. D. Schenck,

M. D.; Treasurer, Alton G. Warner; Member of Executive Committee for three years, J. Lester Keep, M. D.

Among those present were: Drs. Korndorfer and Dudley, Philadelphia; L. A. Phillips, Boston; Mr. Henry Hentz, Ex-Mayor Collins, Mr. Mathews, Mr. and Mrs. Colman, Dr. and Miss Doughty, Dr. and Mrs. Shelton, Dr. and Mrs. Norton, Dr. and Mrs. Porter, Dr. and Mrs. Roberts, Dr. and Miss Paine, Drs. Wilder, Schley, Dennis, Baem, M. Belle Brown, Gaddes, J. V. H. Baker, Cort, Dearborn, Neary, Dr. and Mrs. J. Lester Keep, Drs. Chapin and Warner, Dr. and Mrs. Schenck, and Drs. Paige and Atwood.

